# P27 Establishing an Annual Infection Summit at Leeds Teaching Hospitals NHS Trust – another year, another summit!

**DOI:** 10.1093/jacamr/dlag102.033

**Published:** 2026-06-26

**Authors:** Zara Tariq, Charlie Lobley, Jessica Martin, Emma O’Cofaigh, Kelly Atack, Gillian Hodgson, Kavita Sethi, Sarah Hackney, Lam Nguyen, Shazia Nazir

**Affiliations:** Leeds Teaching Hospitals NHS Trust (LTHT), Leeds, UK; Leeds Teaching Hospitals NHS Trust (LTHT), Leeds, UK; Leeds Teaching Hospitals NHS Trust (LTHT), Leeds, UK; Leeds Teaching Hospitals NHS Trust (LTHT), Leeds, UK; Leeds Teaching Hospitals NHS Trust (LTHT), Leeds, UK; Leeds Teaching Hospitals NHS Trust (LTHT), Leeds, UK; Leeds Teaching Hospitals NHS Trust (LTHT), Leeds, UK; Leeds Teaching Hospitals NHS Trust (LTHT), Leeds, UK; Leeds Teaching Hospitals NHS Trust (LTHT), Leeds, UK; Leeds Teaching Hospitals NHS Trust (LTHT), Leeds, UK

## Abstract

**Background:**

Over 80 colleagues of varying roles, from fourteen different clinical service units (CSU’s) attended including colleagues from community and a patient partner. The morning consisted of educational presentations delivered by AMS experts and local clinical teams championed the achievements made since the inaugural summit in May 2024. The topics included: diagnostic stewardship focusing on urine cultures, our response to difficult to treat infections, managing outbreaks, a patient story ‘experience of a multi-drug-resistant infection,’ reducing inappropriate piperacillin tazobactam in elderly medicine, improving infection prevention and control (IPC) in paediatric haematology/oncology and reducing gram negative bloodstream infections in neonates. The afternoon workshops focused on introducing behaviour change, specifically the COM-B model and how this can used to prevent AMR by improving IPC and antimicrobial prescribing, by focusing on improving capability, motivation and opportunity within teams. This year a follow-up event was held in November 2025, 5 months after the summit in July 2025.

**Objectives:**

To (i) establish an annual educational event led by the infection team for LTHT to collaboratively reduce antimicrobial resistance (AMR); (ii) share and champion progress made in reducing AMR in each clinical area; (iii) collaboratively explore ideas in reducing AMR; and (iv) gather feedback from attendees to improve future summits.

**Methods:**

Post summit, feedback was collected via an electronic, anonymous and optional survey, consisting of quantitative and qualitative questions. The survey concentrated on the content of the event and how attendees felt after the event.

**Results:**

46 colleagues completed the feedback survey; all questions were not mandatory. Increased confidence in using the COM-B model to change behaviour and implement AMS interventions: 39/46. Better understanding of how behaviour change can improve AMS and reduce AMR: 43/46. Ability to contribute to minimizing AMR at LTHT: 44/46. Committed to completing one action post infection summit to minimize AMR: 44/46. Increased knowledge and understanding of AMS and AMR at LTHT: 44/46. Event attendance would lead to improved care to patients: 43/46, Increased empowerment to implement quality improvement ideas locally, to improve AMS and minimize AMR: 42/46. Encouraged to collaborate with other clinical areas: 41/46

**Conclusions:**

The objectives for the summit were achieved successfully with many ideas generated during the afternoon workshops, over 25 AMS improvements implemented post summit (that were highlighted by 6/14 CSU’s) and increased knowledge, empowerment, confidence, commitment and understanding amongst attendees. The summit has allowed the opportunity to collaborate across clinical specialities and professions to align in how we reduce AMR at LTHT. The funding for establishing an annual event has been a limitation however having organized two successful events with lots of progress made, it is essential that we secure funding streams to host another annual infection summit in 2026. Another aspiration 2026 is to host a regional event to encourage sharing progress and collaboration across West Yorkshire in secondary care.

